# Effects of Chitosan Nanoparticles and 4,4′ Methylene-Diphenyl Diisocyanate on the Polylactic Acid/Poly (Butyleneadipate-Co-Terephthalate) Composite Properties

**DOI:** 10.3390/membranes13070637

**Published:** 2023-06-30

**Authors:** Jiaqi Wu, Limei Wang, Bin Qi

**Affiliations:** 1 College of Biology and Food Engineering, Changshu Institute of Technology, Suzhou 215500, China; 20205252013@stu.suda.edu.cn (J.W.); 200600019@cslg.edu.cn (L.W.); 2 College of Pharmaceutical Sciences, Soochow University, Suzhou 215006, China

**Keywords:** polylactic acid, poly (butyleneadipate-co-terephthalate), 4,4′-methylene-diphenyl diisocyanate, chitosan nanoparticles, interfacial compatibility, compatibilizers, composites

## Abstract

Polylactic acid (PLA) is considered a mature alternative to synthetic plastics made from petroleum by-products, possessing the advantages of good mechanical strength. However, it also has some disadvantages such as brittleness and low toughness. In order to overcome and improve some of these unfavorable properties, PLA/PBAT composites were prepared by blending PLA with Poly (butylene adipate-co-terephthalate) (PBAT), and adding 4,4′-methylene diphenyl diisocyanate (MDI) and chitosan nanoparticles (ChNPs) as compatibilizers to investigate the effects of different compatibilizers on the properties of the composites. The main observations are as follows: FT-IR indicated that MDI did not add new groups, while the addition of ChNPs added a substantial amount of hydroxyl and methylene groups. The addition of both MDI and ChNPs did not have any effect on the crystalline shape of the composites, but could potentially reduce their crystallinity, increase the melt peak temperature, wet the boundary of the PLA and PBAT phases, decrease the size of the dispersed phases, reduce the number of dispersed phases, and improve interfacial compatibility. The incorporation of MDI increased the tensile strength from 13.02 MPa to 19.24 MPa, whereas the addition of ChNPs substantially enhanced the elongation at the break from 3.84% to 19.24%. Furthermore, the inclusion of MDI conferred enhanced moisture resistance, whereas the addition of ChNPs seemed to weaken the resistance to moisture.

## 1. Introduction

As the economy continues to grow, the rapid depletion of petroleum reserves due to the widespread use of petroleum-based products and the intensification of greenhouse gas emissions have prompted researchers to explore new materials that are biodegradable, renewable, and recyclable [1]. Biodegradable plastics are plastics that are completely broken down by microbial metabolism in a specific waste treatment system [2]. Biodegradable properties are extremely important in packaging, fibers, and medical stents [3,4,5]. Biodegradable composites represent a viable solution to the challenges posed by waste management and environmental sustainability. Made from a matrix material derived from agricultural and forestry resources, and reinforced with cellulose fibers, these composites offer promise for end-of-life disposal, as they can decompose naturally without harming the ecosystem [6,7]. Compared to traditional petroleum-based synthetic composites, biocomposites have unique advantages such as recyclability, renewability, biodegradability, low density, and affordability, which make them highly attractive for industrial applications [8]. Additionally, biocomposites provide effective sound and thermal insulation, as well as being non-toxic, which reduces the risk of exposure to harmful substances associated with synthetic composites [9].

Biodegradable plastics can be broadly categorized based on their carbon source into two types, namely bio-based biodegradable plastics and petrochemical-based biodegradable plastics [10]. The former group includes starch-based plastics, polylactic acid (PLA), and polyhydroxy fatty acid esters (PHA). The latter group primarily comprises poly(adipic acid/butylene terephthalate) (PBAT) and polybutylene glycol succinate (PBS). PLA, a bio-based alternative to synthetic plastics, enjoys high biocompatibility and excellent tensile strength [11], and is the most extensively produced biodegradable plastic annually [12], making it a widely popular choice. PLA is known for its excellent clarity, bright appearance, high rigidity, and adaptability to various processing conditions [13]. Lactic acid, the building block of PLA, is produced by fermenting a variety of sugars, including glucose, sucrose, and lactose, which can be obtained from various raw materials. Widely regarded as one of the top ten sustainable molecules for the future, lactic acid has the potential for numerous applications in industry [14]. PLA is renewable and biodegradable, with raw materials able to be obtained from crops such as sugar beets and corn. It has good mechanical strength and high stiffness, and its composites can be manufactured by conventional manufacturing methods [15,16].

PLA has excellent tensile properties, but its disadvantages are extreme brittleness [17], elongation at break less than 10%, and low toughness, which greatly limit its applications. To compensate for the disadvantages of PLA, other biodegradable polymers need to be used to improve the physical properties of PLA; among these, PBAT with high toughness and a high elongation at break [18] is considered to be a very promising candidate. PBAT is a synthetic polymer that is derived from fossil resources. However, it is 100% biodegradable and possesses high elongation at the break as well as elasticity [19]. PBAT is versatile and can be utilized in various applications such as packaging materials like garbage bags, food containers, and film packaging, as well as hygiene products such as material for diaper backs and swabs. Moreover, it can also be used in the biomedical field as well as in industrial composting [20]. PBAT is a linear aliphatic-aromatic random copolyester formed through the condensation of 1,4-butanediol, adipic acid, and terephthalic acid. This material can be produced using either fossil or annually renewable resources [21,22]. Polylactic acid has gained favor among numerous researchers owing to its high tensile strength, although its brittleness curtails its extensive application. Conversely, PBAT exhibits high elasticity and crack resistance, but its stiffness and strength are comparatively deficient. When PLA and PBAT act synergistically, biopolymers with significant improvements in processing, mechanical, and thermal properties can be obtained [23,24,25]. However, PBAT/PLA blends have a semi-crystalline structure that makes them difficult to be miscible [26], and the tensile strength and elongation of PBAT/PLA blends are reduced compared to the pure components. Therefore, additives called compatibilizers in polymer blends were developed to improve the melt strength and thermal stability of polymer blends [27].

4,4′-Methylene-diphenyl diisocyanate (MDI) is a widely used compatibilizer in two-component systems based on reactive polyurethane chemistry due to its possession of two isocyanate groups and high reactivity towards carboxyl and hydroxyl groups. Thus, MDI serves to enhance interfacial compatibility. Isocyanate groups readily react with hydroxyl and carboxylic acid groups to form carbamate groups [28]. Pan et al. [28] examined the impact of MDI on the properties of composites by utilizing MDI as a compatibilizer incorporated into the composites.

MDI is a commonly used compatibilizer, but MDI is toxic; we therefore wanted to find a new type of compatibilizer to replace MDI, and after preliminary research, we chose chitosan nanoparticles. Chitosan is a non-toxic, biodegradable, and biocompatible cationic polysaccharide derived from the partial deacetylation of chitin, which is isolated from the exoskeletons of natural crustaceans. Biocompatibility can have a significant impact on the human body as well as the environment [29,30,31], so this property is very important for material selection, It ensures there is no negative response to the issue. It has β 1–4 linked A unit (chitin monomer) and 2-amino 2-deoxy-β-D-glucopyranose (Glc N; D units; chitosan monomer) [32,33,34]. Due to its outstanding biocompatibility, biodegradability, and multifunctional group properties, chitosan has been extensively studied in fields such as biosensors, tissue engineering, separation membranes, and water treatment. Nevertheless, the high cost, subpar mechanical properties, and poor thermal stability of chitosan restrict its application in packaging. Antimicrobial capabilities in some nano- and nanocomposites have been well-established and developed within the packaging industry. Recently developed nano-composite packaging materials possessing antimicrobial functionalities hold significant potential for use as active food packaging materials. Consequently, ChNPs were integrated into PLA/PBAT composites to create films with tensile strength as well as an elongation at break sufficient for an application to subsequent production.

The primary objective of this paper is to contrast the impacts of the commonly utilized compatibilizers MDI with the ChNPs for enhancing interfacial compatibility in PLA/PBAT composites. This will be done by examining changes in the chemical structure, crystalline structure, thermal properties, and mechanical properties resulting from the addition of these agents to the composites. The goal is to analyze the effects of incorporating ChNPs into the composites to provide a reference for future research and production.

## 2. Experimental

### 2.1. Materials

PLA (Ingeo 4032D, degree of polymerization: 200) was purchased from the Nature Works Company (Blair, NE, USA).

PBAT (Mn~120,000) was purchased from the Macklin Biochemical Co., Ltd. (Shanghai, China).

MDI (Purity: 98%) was purchased from the Aladdin Biochemical Technology Co., Ltd. (Shanghai, China).

Chitosan (Deacetylation degree ≥95%, viscosity:100–200 mPa·s) was purchased from the Macklin Biochemical Co., Ltd. (Shanghai, China).

Sodium Tripolyphosphate (AR, purity: 98%) was purchased from the Aladdin Biochemical Technology Co., Ltd. (Shanghai, China).

Trichloromethane (AR) was purchased from the Chinasun Specialty Products Co., Ltd. (Suzhou, China)

### 2.2. Preparation of Chitosan Nanoparticles and Membranes

Chitosan nanoparticles (ChNPs) were prepared using the ionic cross-linking method [35]. Chitosan was dissolved in a 1% acetic acid solution to prepare a chitosan solution with a concentration of 3 mg/mL. A sodium tripolyphosphate solution of 0.8 mg/mL was added to the chitosan solution at a ratio of 3:1 for chitosan: sodium tripolyphosphate. The pH was adjusted to 6.0, and the mixture was stirred at room temperature for 1 h. Following this, the solution underwent centrifugation three to four times, and the precipitate was placed in a freezer at −80 °C for 2 h. Afterward, the sample was freeze-dried for 36 h to obtain ChNPs.

PLA and PBAT composite films were prepared using the solution casting method [36]. PLA and PBAT were added to an appropriate amount of chloroform and stirred at room temperature until completely dissolved. A specific quantity of ChNPs or MDI was weighed and added to the chloroform dispersion containing PLA and PBAT. Subsequently, the mixture was stirred for 1 h at room temperature and sonicated for 30 min to remove air bubbles from the solution. This blend was poured into a PTFE mold and dried in a fume hood at room temperature for 18 h. Finally, the film was peeled off and dried in an oven at 45 °C for 24 h to obtain PLA/PBAT composites with a thickness of (0.18 ± 0.02) mm, suitable for performance testing. Based on prior experiments, the composite material’s mechanical properties will be improved remarkably when 1% of compatibilizer is added. However, its mechanical properties would either be insignificantly enhanced or even diminished if any added volume exceeds the specified quantity. To ensure an equitable proportion of the two volumizing agents, each will be added at a rate of 1%. The specific configuration is shown in Table 1.

### 2.3. Performance Testing and Characterization Methods

#### 2.3.1. Test Equipment

Field emission scanning electron microscope, regulus 8100, Hitachi, Ltd. (Tokyo, Japan).

Differential scanning calorimetry analyzer, DSC8000, PerkinElmer, Ltd. (Shelton, CT, USA).

X-ray powder diffractometer, Rigaku Co., Ltd. (Tokyo, Japan).

Fourier transform infrared microscope system, spotlight 200i, PerkinElmer, Ltd. (USA).

Electronic universal testing machine, UTM4103, SUNS Technology Stock Co., Ltd. (Shenzhen, China)

#### 2.3.2. Thermal Properties Analysis

The crystallinity (*Xc*, %) and melting temperature (Tm) of PLA composites were determined using differential scanning calorimetry (DSC). The samples were heated up to 300 °C and the melt heat absorption curve was recorded. *Xc* was calculated with Equation (1) [37]:

(1) $$X_{c}=\frac{\Delta H_{m}-\Delta H_{c}}{\Delta H_{m^{0}}}\times 100\%\;$$

where $\Delta H_{m}$ is the enthalpy of melting, the unit is J/g, $\Delta H_{c}$ enthalpy of crystallization that occurs during heating; in this case, the value is zero, and $\Delta H_{m^{0}}$ is the equilibrium enthalpy of melting, with a value of 93 J/g.

#### 2.3.3. Infrared Spectral Analysis

Fourier transform infrared spectroscopy (FTIR) was employed for the qualitative identification of components in the composites. The measured wavenumber range spanned from 4000 to 450 cm^−1^ with a resolution of 4 cm^−1^ and a scan number of 64.

#### 2.3.4. Crystalline Structure Analysis

X-ray diffraction (XRD) was utilized for characterizing PLA/PBAT composites, using Cu-Kα rays, a Ni sheet filter, a wavelength of 0.15 mm, a radiation tube voltage of 40 kV, a tube current of 40 mA, a scan rate of 0.02°/s, and a diffraction angle of 2θ ranging from 10° to 60°.

#### 2.3.5. Morphological Analysis

Scanning electron microscopy (SEM) was used to observe the surface and section morphology of PLA/PBAT composites at a magnification of 10^3^× and an acceleration voltage of 1 kV. Additionally, the microscopic morphology of ChNPs was observed by SEM at magnifications between 150× and 10^4^×, and an acceleration voltage of 1 kV.

#### 2.3.6. Mechanical Properties Analysis

Specimens were tested at room temperature using an electronic universal testing machine following GB/T1040.3-2006 Type 1B specifications with a sample length of 70 mm and a tensile rate of 2 mm/min. Each group of specimens was tested three times to obtain the average value.

#### 2.3.7. Water Content and Swelling Analysis

Following the testing method by Zhang Zimo et al. [38], PLA/PBAT composites that measure approximately 1 cm × 1 cm were selected and weighed as m_0_. The samples were then placed in an oven at 90 °C for drying for 24 h, after which they were removed and weighed again as m_1_. Afterwards, the PLA/PBAT composites were immersed in distilled water for 24 h before being taken out to calculate the water content using Equation (2) and the swelling degree using Equation (3):

(2) $$W=\frac{m_{0}-m_{1}}{m_{0}}\times 100\%\;$$

(3) $$S=\frac{m_{2}-m_{1}}{m_{1}}\times 100\%$$

## 3. Results

### 3.1. Thermal Properties Analysis

**Figure 1:**
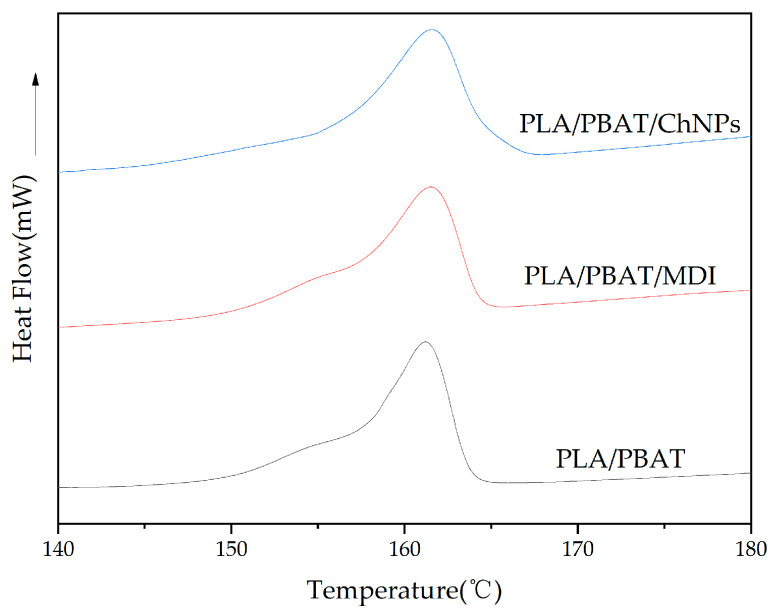
Melting endothermic curve of PLA/PBAT composites prepared with different compatibilizers at a heating rate of 10 °C/min.

Figure 1 displays the non-isothermal crystallization curves of the composites obtained by incorporating MDI [39] and ChNPs into PLA/PBAT composites, with a heating rate of 10 °C/min from 30 °C to 300 °C. Both the PLA/PBAT composites and those with various compatibilizers exhibit analogous thermal characteristics. Each composite displayed a single broad peak corresponding to the melting temperature of the blends, signifying improved dispersion between blend constituents.

In comparison to the PLA/PBAT composites, the melting peaks of the composites containing compatibilizers demonstrated a propensity to shift to the right in the melting curves. Possibly, some hydroxyl groups and end groups in PLA participated in the chain elongation reaction, and the chain expansion through MDI addition augmented the molecular weight while reducing the number of end groups [40]. Furthermore, the incorporation of rigid aromatic structures impeded chain mobility [28].

Following the addition of ChNPs, the melting peak increased from 161.21 °C to 161.50 °C (Table 2). This alteration could be ascribed to the interaction between the hydroxyl group of ChNPs and the ester group of PBAT, which is consistent with the Tm increase observed by Sliva et al. [41] for PBAT blends utilizing MA as compatibilizers. The crystallinity of each composite, calculated from the melt enthalpy and the crystallinity equation, reveals that the introduction of compatibilizers slightly diminishes the crystallinity compared to the PLA/PBAT composites: from 18.03% to 16.78% with MDI addition, and from 18.03% to 17.12% with ChNPs addition. The inclusion of chitosan nanoparticles, akin to MDI, lowered the composites’ crystallinity. This finding establishes that ChNPs can serve as compatibilizers, enhance interfacial compatibility between PLA and PBAT, and reduce the crystallinity of PLA/PBAT/ChNPs composites—conclusions corroborated by SEM results.

### 3.2. Infrared Spectral Analysis

**Figure 2:**
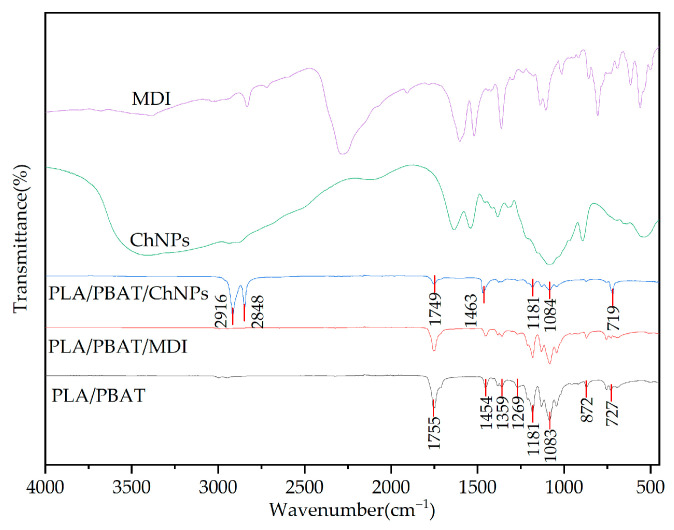
FT-IR spectra of PLA/PBAT composites prepared with different compatibilizers and the compatibilizers.

Figure 2 displays the FTIR spectra of PLA/poly (butylene terephthalate-adipate) (PBAT) composites and composites containing added compatibilizers. The PLA/PBAT composites exhibit a distinct absorption peak at 1755 cm^−1^, attributable to the stretching vibration of C=O, and another distinct peak at 1454 cm^−1^ caused by the distortion of the hydroxyl group; both peaks are typical of PLA transmission bands. Asymmetric vibrations of the ester group in PLA can also be observed at 1269 cm^−1^ and 1181 cm^−1^ [42,43,44]. The absorption peak at 1269 cm^−1^ corresponds to the C=O stretching peak of carbonyl and ester groups, which is typical of the IR transmission band of PBAT [43]. Moreover, peaks at 1359 cm^−1^, representing a symmetric stretching vibration of -COOH, a characteristic C-O-C absorption peak around 1083 cm^−1^, and a peak at 872 cm^−1^, signifying a C-H bending peak, can be observed. The peak at 727 cm^−1^ is the methylene stretching peak [45].

**Scheme 1:**
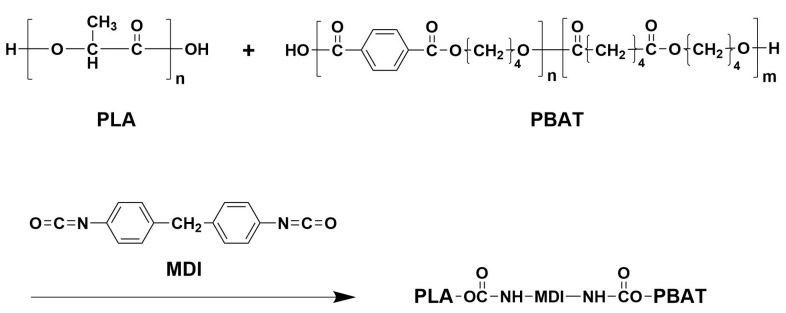
Reaction of MDI with PLA and PBAT.

Clearly, the characteristic peaks did not change significantly after the addition of MDI as a bulking agent, and no stretching band corresponding to the isocyanate group was found at 2270 cm^−1^ [28], indicating that the initial isocyanate group reacted completely with PLA and PBAT without introducing new groups. The reactions are shown in Scheme 1.

Conversely, upon incorporating chitosan nanoparticles as compatibilizers, it can be observed that although most of the characteristic peaks maintained their positions, the intensities of the peaks altered. The C=O stretching peak at 1755 cm^−1^, symmetric stretching vibration peak of -COOH around 1360 cm^−1^, asymmetric vibration peaks of the ester group at 1270 cm^−1^ and 1181 cm^−1^, C-O-C characteristic absorption peak at 1083 cm^−1^, and interstitial substitution peak of the benzene ring at 872 cm^−1^ all decreased in intensity. Simultaneously, the distorted peak of the hydroxyl group at 1460 cm^−1^ and the telescopic peak of the methylene group at 720 cm^−1^ experienced an increase in intensity [35,46]. Notably, new high-intensity telescopic peaks of -CH2 at 2916 cm^−1^ and 2848 cm^−1^ were distinctly observed. Miao et al. [47] stated that chitosan nanoparticles possess abundant hydroxyl groups in their chains, which can interact with polymer chains due to a strong hydrogen bonding force. As a result, the interaction between PBAT/PLA and ChNPs may facilitate the formation of rigid networks between them. Therefore, incorporating chitosan nanoparticles can effectively enhance the interactions between biocomposite components and improve interfacial compatibility.

### 3.3. Crystallographic Structure Analysis

**Figure 3:**
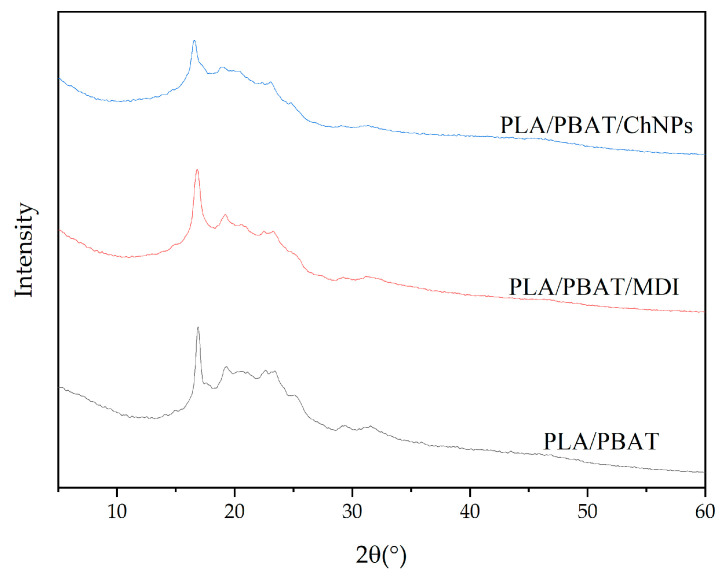
X-ray diffraction patterns of PLA/PBAT, PLA/PBAT/MDI and PLA/PBAT/ChNPs composites.

Figure 3 presents the X-ray diffraction patterns of the PLA/PBAT composites after adding MDI and ChNPs. The incorporation of these reinforcing agents did not change the positions of the diffraction peaks, which remain evident at 2θ = 16.9°, 19.3°, 22.4°, and 23.4°. The diffraction peak at 2θ = 16.9° corresponds to the (110) or (200) crystal plane of α-type crystals, while the peak at 2θ = 19.3° is associated with the α-type crystals of the (111) crystal plane. Similarly, the diffraction peaks at 2θ = 22.4° and 23.4° correspond to the α-type crystals of the (210) and (112) crystal planes, respectively. Thus, it is evident that the crystalline phase of the composite is of an α crystal type.

After incorporating MDI and ChNPs, both the positions and numbers of diffraction peaks in the composites remained unchanged; however, the intensity of the peaks diminished. Notably, the intensity of the diffraction peak at 2θ = 16.9° decreased significantly after adding ChNPs [48,49]. This observation suggests that while the introduction of reinforcing agents does not alter the crystalline structure of the PLA/PBAT composites, it does result in a decrease in crystallinity. This finding is consistent with DSC results and may be attributed to strong hydrogen bonding induced by the abundant hydroxyl groups in ChNPs, which enhances the interfacial compatibility between PLA and PBAT, consequently leading to reduced crystallinity.

### 3.4. Microscopic Morphology

**Figure 4:**
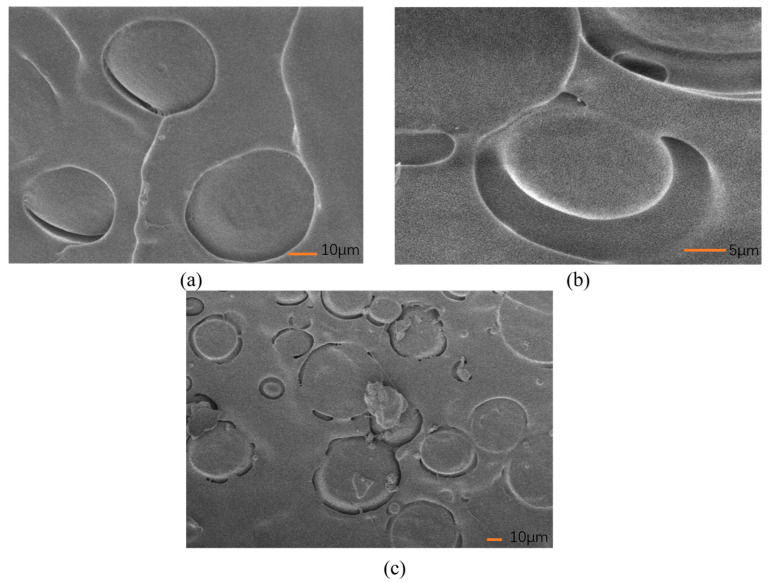
SEM image of composite material cross-section: (**a**) PLA/PBAT with a magnification of 10,000; (**b**) PLA/PBAT/ChNPs with a magnification of 30,000; (**c**) PLA/PBAT/MDI with a magnification of 5000.

Figure 4 displays the cross-sectional SEM images of the composite PLA/PBAT and the composites containing various compatibilizers. The distinct “sea-island” phenomenon of the dispersed phase (PBAT) is observable in Figure 4a. As depicted in the figure, there is virtually no wetting between the PBAT and PLA phases, demonstrating low compatibility. For the PLA/PBAT blend, a smooth debonding surface is present, signifying a complete phase separation.

After increasing the magnification, compared to Figure 4a where the dispersed phase appears as a complete circle, Figure 4b shows a significant decrease in the area of the dispersed phase and a wetting of the boundary of the dispersed phase, and Figure 4c also shows a significant blurring of the boundary of the dispersed phase, which is no longer a separate “island” and is connected to another phase. This also demonstrates that ChNPs and MDI can act as compatibilizers to enhance the interfacial compatibility of PLA and PBAT.

**Figure 5:**
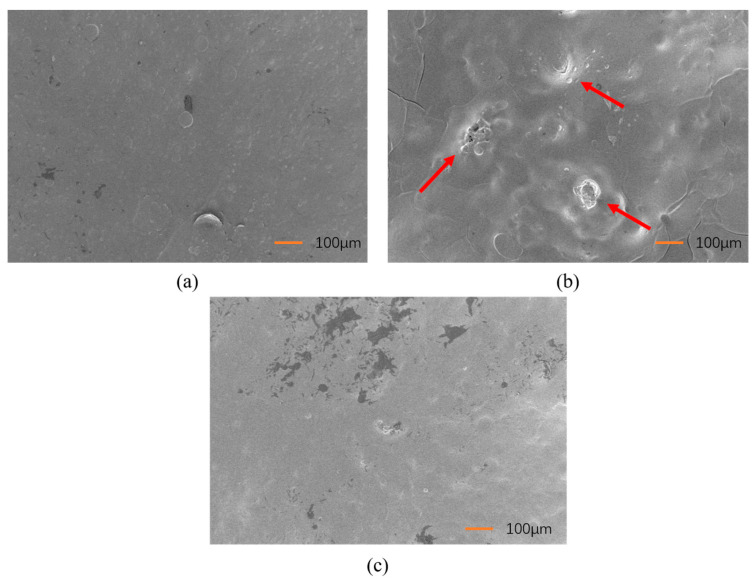
SEM image of composite material surface:(**a**) PLA/PBAT with a magnification of 1000; (**b**) PLA/PBAT/ChNPs with a magnification of 1000; (**c**) PLA/PBAT/MDI with a magnification of 1000.

Figure 5 exhibits the surface SEM images of the composite PLA/PBAT as well as PLA/PBAT/ChNPs and PLA/PBAT/MDI. It is evident that membranes made from both PLA and PBAT possess smooth and flat surfaces, and remain intact and continuous after incorporating the compatibilizers. Nonetheless, the addition of chitosan nanoparticles generates protrusions on the membrane surfaces; these result from the entanglement of ChNPs within the composites. Electron microscopy reveals that ChNPs are uniformly distributed inside the matrix without any pores, indicating compatibility between the composites and ChNPs. The absence of pores signifies excellent compatibility and interfacial adhesion between the composites and ChNPs. Hydrogen bonding interactions between the ester groups of PLA/PBAT and the hydroxyl and amino groups of chitosan nanoparticles enhance the compatibility between the matrix and the compatibilizer.

### 3.5. Mechanical Properties Analysis

Polyester-based composites are widely used in flexible packaging applications, which require a good balance between flexibility and strength. For this purpose, information on the static mechanical properties of PLA/PBAT and composites with different compatibilizers added to PLA/PBAT were obtained by tensile tests, and the tensile strength and elongation at break of the materials are shown in Table 3. The tensile strength of PLA/PBAT/MDI composites was 13.02 MPa with an elongation at break of 3.84%, while the tensile strength of PLA/PBAT/MDI composites was 19.24 MPa and the elongation at break was 4.37%. It can be observed that the addition of MDI increased the tensile strength by 47.1%, and there was also a small increase in the elongation at break due to the addition of MDI as a bulking agent, resulting in enhanced interfacial adhesion. The relative molecular mass of PLA and PBAT increased, improving the tensile strength and elongation at break.

Interestingly, the addition of chitosan nanoparticles (ChNPs) to the composites as a bulking agent had little effect on the tensile strength but significantly increased the elongation at break of the composites to 19.24%, with a tensile strength of 13.20 MPa. This is due to the fact that the tensile strength of the composites is mainly provided by PLA, and in the PLA/PBAT composites, the interfacial compatibility between PLA and PBAT is poor, with PLA being dominant. After the addition of ChNPs, the elongation at break reached 19.24%, an increase of 401%. The addition of ChNPs can significantly enhance the high toughness of PBAT and greatly improve the elongation at break of the composite due to ChNPs as a bulking agent enhancing the interfacial compatibility between PLA and PBAT, reducing the dispersed phase, promoting their interaction, and increasing the strength of the blended interface with a large amount of hydrogen bonding and entanglement.

### 3.6. Analysis of Water Resistance

The water content and swelling of a membrane are dependent on the presence or absence of hydrogen bonding, ionization of amino or carboxyl groups, and re-relaxation of the membrane structure. These factors reflect the membrane’s ability to resist moisture from external sources [38]. As indicated in Table 4, the PLA/PBAT film had a water content of 3.95% and swelling of 1.37%. Both the water content and swelling were reduced upon adding MDI as a compatibilizer. This decrease was due to the reaction between MDI and the hydrogen bonds present at both ends of PLA and PBAT, resulting in a reduction in hydrogen bonds in the film and an increased ability to resist external moisture. In contrast, the addition of ChNPs increased the water content and swelling because ChNPs contain numerous hydrogen bonds, which reduced the film’s ability to resist moisture by introducing more hydrogen bonds. This difference may also explain why there are variations in mechanical properties between the PLA/PBAT/ChNPs and PLA/PBAT/MDI films.

## 4. Conclusions

In this paper, the effects of MDI and ChNPs addition on the chemical structure, crystallization behavior, thermal properties, microscopic morphology, mechanical properties, and resistance to moisture of PLA/PBAT composites were investigated. The FTIR spectra demonstrated that the starting isocyanate groups reacted completely with PLA and PBAT, without adding any new groups. In contrast, the addition of ChNPs introduced a substantial number of -CH2 groups as well as hydroxyl groups. The addition of both MDI and ChNPs did not change the crystalline shape of the composites, but the crystallinity of PLA/PBAT/MDI and PLA/PBAT/ChNPs decreased from 18.03% to 16.78% and 17.12%, respectively. The addition of both substances was able to promote wetting at the boundary of the PLA and PBAT phases, reduce the size of the dispersed phases, and decrease their number. This confirmed that MDI and ChNPs could effectively improve interfacial compatibility. It was also observed that both components were uniformly distributed within the matrix without pores, indicating good compatibility and interfacial adhesion between the composites and ChNPs or MDI. Both MDI and ChNPs can enhance the mechanical properties of the composites. However, the addition of MDI primarily increases the tensile strength from 13.02 MPa to 19.24 MPa, while the addition of ChNPs mainly raises the elongation at break from 3.84% to 19.24%. The incorporation of MDI reacts with the -OH at both ends of PLA and PBAT, resulting in reduced hydroxyl groups in the composites, which leads to improved resistance to moisture. In contrast, the addition of ChNPs introduces a large quantity of hydroxyl groups, making the composites less resistant to moisture.

From the experimental results, it is easy to see that although they are both compatibilizers, MDI and ChNPs have different effects on the chemical structure, mechanical properties and moisture resistance of the materials. We are studying the specific reactions of both in the materials to further investigate whether ChNPs as compatibilizers can further improve the performance of the composites and provide a good modification solution for PLA/PBAT composites. However, further studies are needed to determine the feasibility of applying these materials in industrial production.

## Figures and Tables

**Table 1:** Ratio of PLA/PBAT composites.

Samples	PLA (wt%)	PBAT (wt%)	Compatibilizers (wt%)
PLA/PBAT	50	50	0
PLA/PBAT/MDI	49.5	49.5	1
PLA/PBAT/ChNPs	49.5	49.5	1

**Table 2:** Tm, ΔHm and Xc of different composite materials.

Samples	Tm (°C)	ΔHm (J/g)	Xc (%)
PLA/PBAT	161.21	16.77	18.03
PLA/PBAT/MDI	161.52	15.61	16.78
PLA/PBAT/ChNPs	161.50	15.83	17.12

**Table 3:** Mechanical properties of PLA/PBAT composites prepared with different compatibilizers.

Sample	Tensile Strength (Mpa)	Elongation at Break (%)
PLA/PBAT	13.02 ± 0.83	3.84 ± 0.20
PLA/PBAT/MDI	19.24 ± 1.14	4.37 ± 0.43
PLA/PBAT/ChNPs	13.20 ± 0.51	19.24 ± 1.35

**Table 4:** Water content and swelling degree of PLA/PBAT composites prepared with different compatibilizers.

Sample	Water Content (%)	Swelling Degree (%)
PLA/PBAT	3.95%	1.37%
PLA/PBAT/MDI	1.71%	0.09%
PLA/PBAT/ChNPs	6.69%	2.62%

## Data Availability

Not applicable.
